# Single DNA Origami Detection by Nanoimpact Electrochemistry

**DOI:** 10.1002/celc.202101696

**Published:** 2022-02-17

**Authors:** Evangelina Pensa, Yash Bogawat, Friedrich C. Simmel, Ibon Santiago

**Affiliations:** ^1^ Physics Department and ZNN Technische Universität München Am Coulombwall 4a 85748 Garching Germany

**Keywords:** DNA nanotechnology, DNA origami, Electrochemistry, Methylene blue, Nanoimpact

## Abstract

DNA has emerged as the material of choice for producing supramolecular building blocks of arbitrary geometry from the ‘bottom up′. Characterisation of these structures via electron or atomic force microscopy usually requires their surface immobilisation. In this work, we developed a nanoimpact electrochemistry platform to detect DNA self‐assembled origami structures in solution, using the intercalator methylene blue as a redox probe. Here, we report the electrochemical detection of single DNA origami collisions at Pt microelectrodes. Our work paves the way towards the characterisation of DNA nanostructures in solution via nanoimpact electrochemistry.

## Graphical abstract

**Collision course**: Collision of DNA origami structures interacting with methylene blue redox intercalator produces a readable current spike due to the MB reduction, allowing the detection of single DNA‐origami.

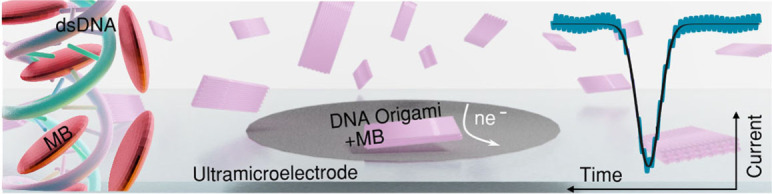

## Introduction

DNA is a remarkable material for building supramolecular structures of almost arbitrary geometry from the ‘bottom up’, offering improved precision in the rational design of nanostructures. Structural DNA nanotechnology has progressed tremendously in recent years and has facilitated the formation of intricate nanostructures extended in two and three dimensions with near‐atomic precision using the self‐assembly of DNA strands whose interactions are programmed through the design of their base sequences. Among these techniques, DNA origami technology has shown particular utility in the bottom‐up fabrication of well‐defined nanostructures ranging from tens to hundreds of nanometres.^[1]^ Typically a 7‐kbase DNA scaffold strand is folded into a structure by hybridisation to hundreds of synthetic ‘staple’ oligonucleotides, which allows diverse structures to be formed.^[2]^

Transmission electron microscopy (TEM),^[1a]^ fluorescence microscopy^[3]^ and atomic force microscopy (AFM)^[1b]^ are often used techniques for the characterisation of DNA origami structures. Fluorescence methods, however, require appropriate modification of the structures. Further, many methods require surface immobilisation of the structures and are thus rather static and involve the use of sophisticated and expensive apparatuses. While these tools allow the physical characterisation of the shape and size of DNA structures, they do not offer a direct way of detecting them in solution, which could be advantageous for biosensing applications.

Particle‐electrode collisions (also known as nanoimpact) is a rapidly growing research area which aims at the in‐situ direct detection of single nanoparticles in solution.^[4]^ This method has been used with inorganic and organic nanoparticles,^[5]^ emulsion droplets,^[6]^ lipid vesicles,^[7]^ bacteria,^[8]^ viruses and enzymes,^[9]^. It has also been utilised as a biosensor to indirectly detect tumour biomarkers^[10]^ and viral DNA.^[11]^ However, so far, little work has been focused on the detection of DNA self‐assembled nanostructures. The main reason is that DNA is not electrochemically active in terms of current density and reversibility.^[12]^ The electrochemical detection of DNA usually involves modifying oligonucleotides with redox moieties^[13]^ or a multiple‐step modification of the electrode surface with oligonucleotides.^[14]^

**Figure 1:**
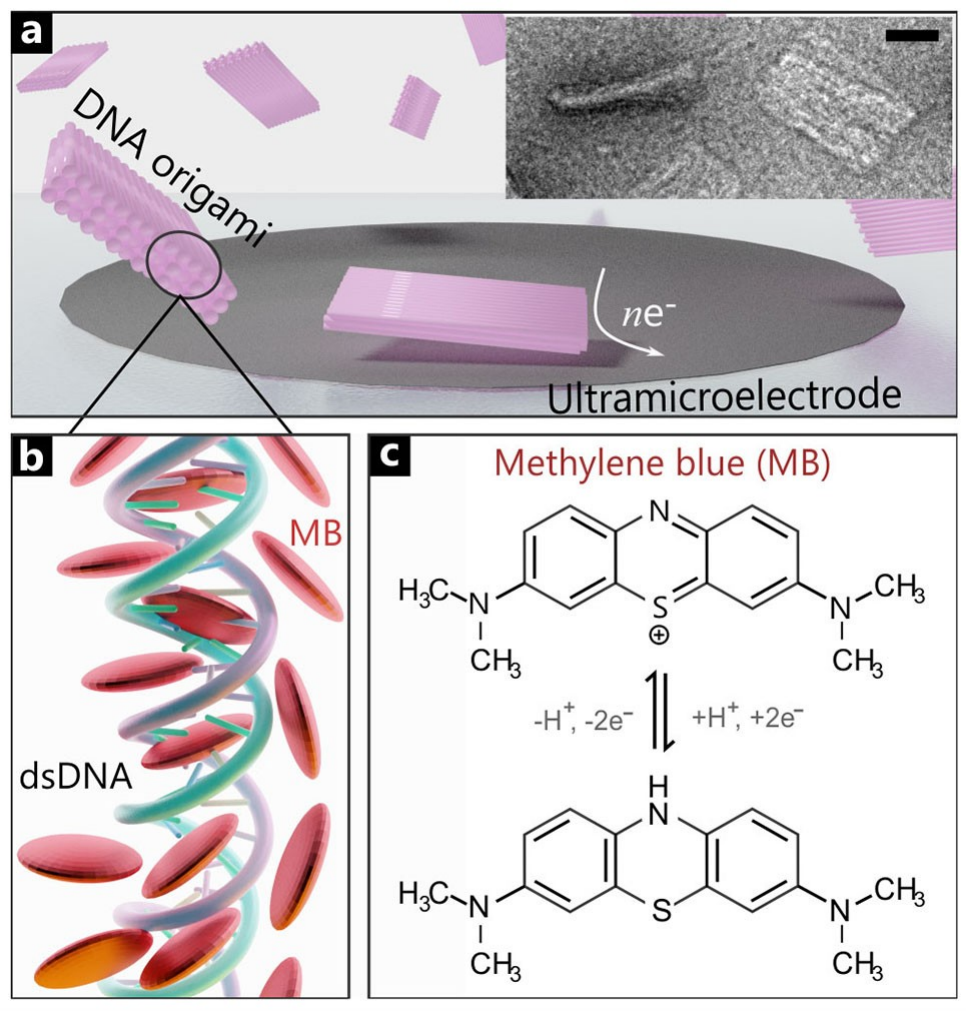
a) Scheme of DNA origami monolith structure collisions on a Pt ultramicroelectrode. Inset: TEM Image of MB‐Origami sample. Scale bar corresponds to 20 nm. b) Interaction between methylene blue (MB) molecules and double‐stranded DNA (dsDNA). c) Reduction and oxidation reactions of methylene blue.^[16]^

In this work, we develop a nanoimpact electrochemistry platform to detect DNA self‐assembled origami structures in solution without introducing extra elements into the DNA origami or surface modification of the electrode. We produced a simple brick‐shaped origami structure (shown in Figure 1a) and rendered it redox‐active using methylene blue (MB), which is an electrochemically active dye and has been widely used in biochemistry as a DNA intercalator. DNA intercalating molecules with therapeutic applications like doxorubicin have been used on DNA origami structures for tunable targeted drug delivery.^[15]^ The impact of DNA origami structures on the microelectrode results in current spikes, which scale with the origami concentration in solution. This work opens the door to the direct detection of DNA origami using nanoimpact electrochemistry.

## Results and Discussion

We synthesised a rigid 3D DNA origami block (molecular weight 5.2 MDa), following the design principles outlined in Ref. [17] and as described in detail in the Supporting Information (SI‐1). The rigid block (hereafter referred to as ‘monolith’) comprises three layers of 14 DNA helices tightly packed in a square lattice and held together by oligonucleotide crossovers. Each helix bundle is 200 base pairs in length (the DNA caDNAno file and list of staples are included in SI‐1). TEM and AFM micrographs of the resulting DNA bricks are shown in SI‐2.1 and SI‐2.2, respectively: their approximate dimensions measured by AFM are width 60 nm, depth 6 nm and length 36 nm. SI‐2.3 shows UV characterisation of the monolith structure. Correct folding of the structures was assessed using an agarose gel shift mobility assay (SI‐2.4). The TEM micrographs (inset in Figure 1a) and agarose gel shift mobility assay rule out aggregation of DNA origami in the presence of MB. Further, the intercalation of MB does not alter the structural integrity of DNA origami.

Because of the lack of redox activity of DNA, we supplied the monolith with electrochemical activity using the redox‐active intercalator methylene blue. This molecule comprises planar aromatic rings, which can intercalate between the base pairs of DNA (Figure 1b). At physiological pH, MB can undergo a two‐electron/one proton transfer oxidation/reduction (Figure 1c).

**Figure 2:**
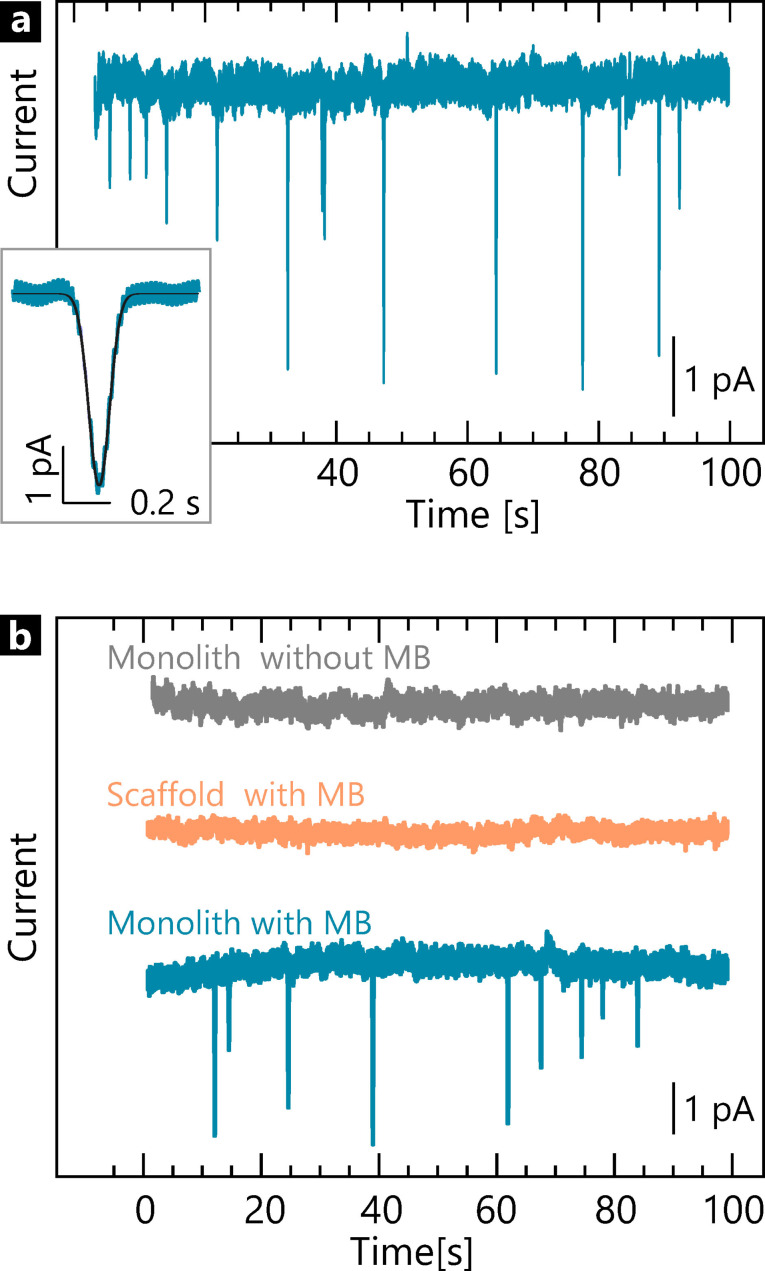
a) Spikes observed in a representative i‐t curve at Pt‐ultramicroelectrode immersed in a solution containing monolith and 10 μM MB in TBE buffer; CA traces were recorded at −0.3 V with a sampling interval of 100 μs. Inset shows a single spike. b) Control experiments of monolith in solution in the absence of MB (top) and scaffold strand (mid) and monolith (bottom) in the presence of MB.

In order to observe single‐monolith impacts, we carried out measurements using a Pt ultramicroelectrode in TBE buffer containing 10 μM methylene blue (cf. SI‐3). Chronoamperometry (CA) traces were recorded at −0.3 V with a sampling interval of 100 μs. The working potential −0.3 V is chosen so that the reduction of MB takes place (SI‐7.2). A typical i‐t curve is shown in Figure 2a. A representative spike in Figure 2a‐inset shows a charge amount of 9 pC with a height of 3 pA. The charge values of most spikes lie between 0.1 and 9 pC.

**Figure 3:**
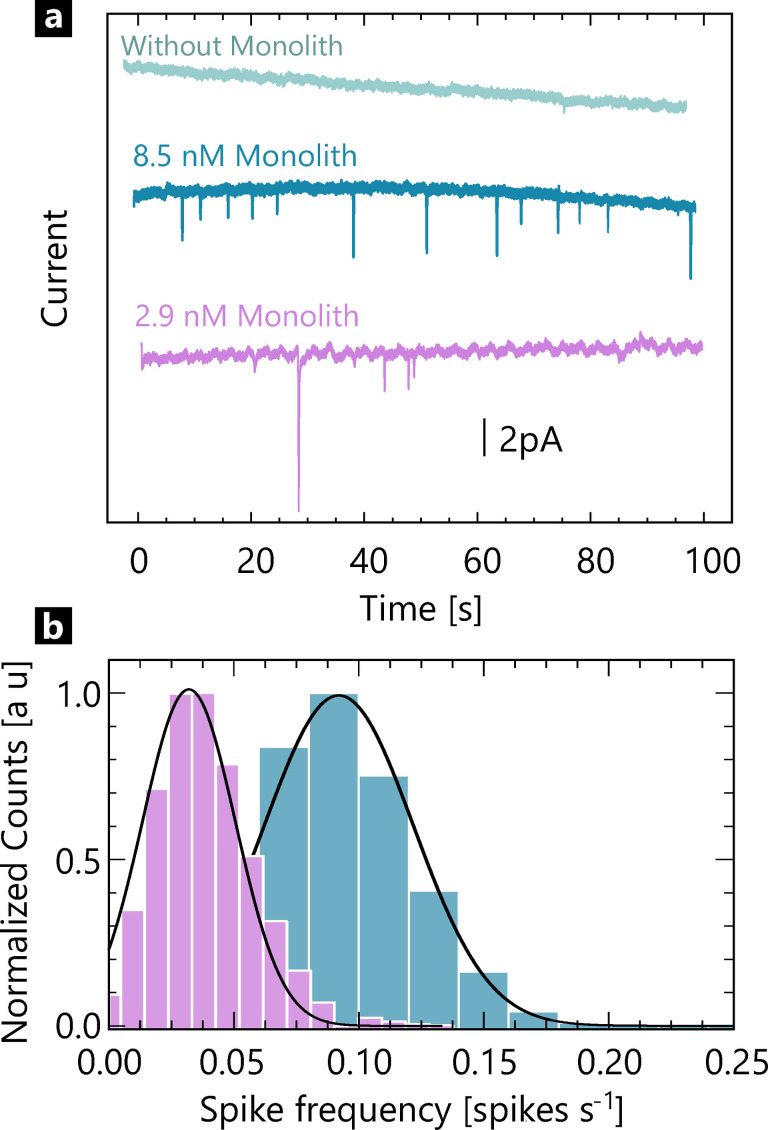
a) i‐t traces at two different monolith concentrations at the same MB concentration. b) Spike histogram showing the average number of spikes per s for 2.9 nM and 8.5 nM DNA monolith.

To confirm that the observed spikes are due to single monolith impacts, we carried out control experiments in the absence of origami and MB, respectively (Figure 2b). No visible spikes were detected in the absence of origami or MB, while spikes appeared when adding 2.9 nM origami. With a higher concentration (8.5 nM), the number of spikes tripled within the same time window of 100 s (Figure 3a). Although MB in the solution is reduced at the electrode and contributes to a background current, it does not cause any observable spikes (SI‐4). Further experiments of adding scaffold DNA (single‐stranded viral DNA derived from bacteriophage M13mp18) or residual PEG polymer showed no significant spikes (SI‐4), indicating that neither contributes to spike formation. The negative control measurements support the hypothesis that the spikes are due to the onolith‐MB collision on the electrode surface in the presence of MB and not due to non‐faradaic processes.

We identified current spikes using a peakfinder algorithm (cf. SI‐5), applying a current threshold to a smoothened raw signal and then counting zero‐point crossings in the first derivative of the signal. Figure 3b shows the average number of spikes per s for 2.9 nM and 8.5 nM DNA monolith, respectively. We measured an average of 0.03 s^−1^ (standard error of the mean 0.02) for 2.9 nM origami and spike frequency of 0.09 s^−1^(standard error of the mean 0.03) for 4 nM.

There are two main DNA binding modes for MB: (i) intercalation between base pairs and (ii) electrostatic interaction between MB and negatively charged DNA phosphate backbone.^[16]^ Considering these mechanisms, we estimated an upper limit for the number of MB molecules bound to a monolith and calculated the expected charge of MB reduction per monolith to be 2.58 fC (details of the calculation are given in SI‐6). Nanoimpact electrochemistry is a sensitive method to detect single particles which are capable of transferring charges to the electrode at the picoCoulomb (pC) level, corresponding to a detectable current signal in the picoAmpere (pA) range.^[9a]^ This theoretical estimate, which only considers DNA intercalation of MB, suggests that nanoimpact electrochemistry would unlikely detect single monoliths, as the expected signal falls below the detection limit.

**Figure 4:**
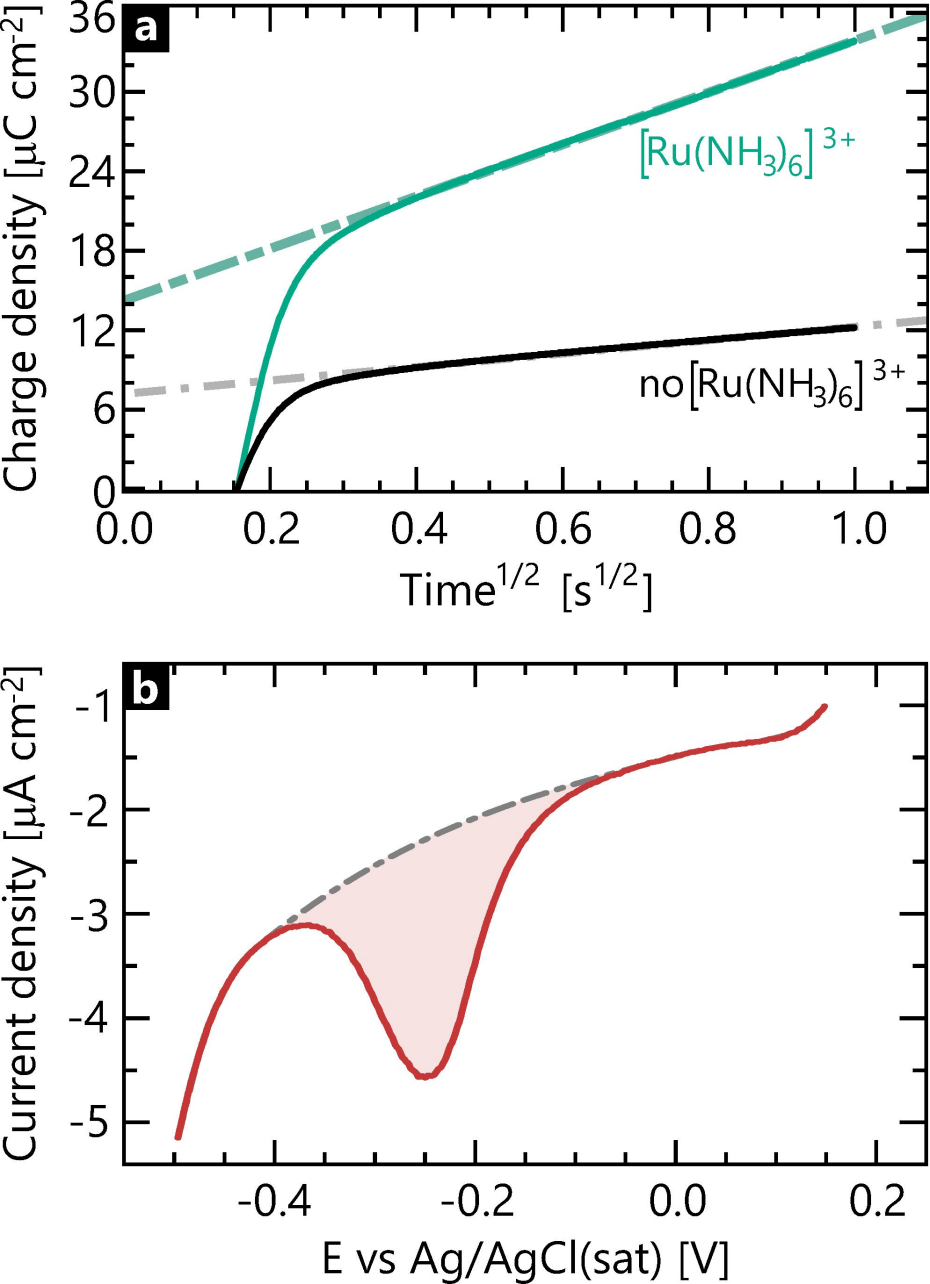
a) Typical charge density *vs* square root of time curves obtained for Au‐monolith samples without (black) and with (green) Ru(NH_3_)_6_Cl_3_. The curves were obtained in 10 mM Tris at −0.3 V, using a pulse period of 1 s. Fit parameters for the linear trend are A =14.3 μC cm^−2^, B=19.5 μC cm^−2^ s^−1/2^, R^2^=0.9997 and A=7.3 μC cm^−2^, B=5.1 μC cm^−2^ s^−1/2^, R^2^=0.998 for samples with and without 50 μM [Ru(NH_3_)_6_]^3+^, respectively. b) Typical cyclic voltammogram obtained for Au‐monolith‐MB samples. The red highlighted area is used to calculate the MB surface coverage. Scan rate: 0.1 V s^−1^, electrolyte: TBE.

To test this hypothesis, we measured the amount of the monolith adsorbed on a Au macroelectrode. To this end, the monolith was deposited on the electrode surface. The electrochemical behaviour of MB intercalated DNA origami is shown in Figure S‐11 with a reduction potential of −0.25 V. The DNA origami coverage was determined by coulometry in the presence and absence of the redox marker ruthenium(III) hexamine, [Ru(NH_3_)_6_]^3+^, as shown in Figure 4a (cf. SI‐7). The coverage is calculated to be (3.3±0.2)×10^10^ origami structures per cm^2^, which is in good agreement with the expected number when the surface is fully saturated (the electrochemical measurement and calculation can be found in SI‐7).

We then immersed the monolith‐coated Au macroelectrode in a 1 mM MB solution and performed cyclic voltammetry in TBE buffer. The cyclic voltammograms of the Au‐Monolith‐MB samples shown in Figure 4b suggest that around 300 MB molecules are incorporated in a single origami structure. This number of MB molecules is equivalent to a charge of 0.1 fC per monolith (cf. SI‐7), a value lying in the same range as the above theoretical estimate, and thus, more importantly, a value that falls below the detection limit for nanoimpact electrochemistry. The nanoimpact signal measured is significantly much larger and thus cannot be understood simply via this estimate. Therefore, other mechanisms might play a role in the generation of the signal.

For nanoimpact of nanoscale species such as proteins, a direct electron transfer has been reported.[^9a^, ^18^] This direct electron transfer from the redox solution to the electrode mediated by the proteins resulted in a three‐fold enhanced current of the spikes. The discrepancy has been explained considering that proteins temporarily act as a nanoelectrode in a diffusion‐controlled or near diffusion‐controlled reaction also referred to as ‘direct electrochemistry’.^[18a]^ We here propose that a similar mechanism takes place for the occurrence of current spikes, whereby the generated charge transfer between electrode and MB solution is mediated directly by the DNA origami in contact with the electrode surface during a nanoimpact event. When nanoimpact occurs, MB is reduced to its charge‐neutral form and desorbs from the negatively charged origami, exposing negatively charged adsorption sites on the origami, thereby attracting more MB from the solution. We can thus regard the origami structure as a drain for the MB molecules in solution near the electrode, which will be adsorbed at the origami and subsequently reduced. This iterative cyclic process continues so long as the origami remains near the electrode, thereby acting as a temporary nanoelectrode. Also, such a nanoelectrode might facilitate a fast charge transfer, contributing to the observed large spike current. Further investigation into redox‐active DNA origami structures can shed light on the mechanism involved in this ‘direct’ electrochemistry mechanism between electrode and origami.

## Conclusion

We developed a simple method utilising electrochemistry to detect self‐assembled origami nanostructures in solution. Without introducing extra elements into the DNA origami or surface modification of the electrode, we can detect collision events of DNA monolith structure with a Pt microelectrode. To activate the DNA monolith electrochemically, we introduce MB into the solution as a redox probe, which interacts with DNA via intercalation. We report the first experimental observation of collision events of DNA origami on a microelectrode in the presence of MB. As we increase the concentration of DNA origami, we observe an increase in collision frequency, while the amplitude remains in the same current range. We propose that the mechanism involves the direct charge transfer between electrode and redox solution mediated by DNA origami. Ultrasensitive electrochemical sensors that utilise DNA origami as a nanoelectrode can be envisaged. For instance, our approach should allow the detection of sensing events that localise the origami to the electrode, where it acts as an antenna for MB molecules. Further, this work paves the way towards the detection of more complex DNA nanostructures using nanoimpact electrochemistry.

## Experimental Section

All chemicals were used as received. Source and purity are indicated in the SI.

*Electrochemical setup*: Electrochemical experiments were performed in a conventional three‐electrode glass cell using a HEKA PG340 USB potentiostat (HEKA, Germany). The electrolyte was deoxygenated thoroughly using nitrogen prior to use, and a blanket of nitrogen was maintained over the solution during all experiments. All measurements were carried out at room temperature (∼25 °C). For all the reported values, the stated errors are (Bessel corrected) standard deviations based on at least 3 independent measurements. Pt coil and a Ag/AgCl(sat) served as the counter electrode and reference electrode, respectively. Au macroelectrodes (IJ Cambria, UK) and Pt ultramicroelectrodes (PtUME, IJ Cambria, UK, CHI107P) were employed as working electrodes (WEs) in bulk electrochemistry and nanonimpact electrochemistry, respectively. Prior to use, WEs were cleaned as described in SI‐3.1.

*Nanoimpact setup*: Nanoimpact measurements were carried out in TBE buffer containing 10 μM methylene blue (MB) at pH=8. Chronoamperometry (CA) traces were recorded at −0.3 V with a sampling interval of 100 μs, a 100 Hz Bessel filter and post digital filter of 3 Hz. Before adding the origami, at least three CA traces in 10 μM MB‐TBE were recorded. These traces do not show any noticeable feature apart from a constant current baseline. Afterwards, an aliquot of the monolith stock solution was added into the cell. The solution was mixed, N_2_ was bubbling in for 5 min and CA traces were recorded.

*DNA origami annealing and purification*: 50 nM of p8540 DNA scaffold, a staple mix containing 380 nM of each DNA staple, were mixed in a folding buffer containing 5 mM Tris, 0.5 mM EDTA, 20 mM MgCl_2_ and 5 mM NaCl, pH=8. Mixtures were annealed and purified using the PEG purification method as described in SI‐1.3. The concentration of DNA origami was measured by UV‐Vis spectroscopy (NanoPhotometer Pearl, Implen GmbH) considering an extinction coefficient of 0.13 nM^−1^ cm^−1^. DNA origami structures were characterised by TEM, AFM and UV‐Vis spectroscopy as described in SI‐2.

## Conflict of interest

The authors declare no conflict of interest.

1

## Supporting information

As a service to our authors and readers, this journal provides supporting information supplied by the authors. Such materials are peer reviewed and may be re‐organized for online delivery, but are not copy‐edited or typeset. Technical support issues arising from supporting information (other than missing files) should be addressed to the authors.

Supporting Information

## Data Availability

The data that support the findings of this study are available from the corresponding author upon reasonable request.
