# Supplementary material for: Single DNA Origami Detection by Nanoimpact Electrochemistry
Source: ChemElectroChem. 2022 Feb 17;9(4):e202101696. doi: 10.1002/celc.202101696 (PMC9302979; doi:10.1002/celc.202101696)
Supplement: Supplementary file 1 — Supporting Information [file CELC-9-0-s001.pdf]

ChemElectroChem

Supporting Information

Single DNA Origami Detection by Nanoimpact Electrochemistry

Evangelina Pensa,* Yash Bogawat, Friedrich C. Simmel, and Ibon Santiago*

CONTENTS

1 Synthesis and purification of Monolith ... 3
1.1 Staple list ... 3
1.2 DNA Origami design ... 7
1.3 Annealing and purification ... 7
2 Characterization of Monolith ... 8
2.1 TEM ... 8
2.2 AFM ... 8
2.3 UV-Vis ... 9
2.4 Agarose Gel Electrophoresis ... 9
3 Material and Methods ... 10
3.1 Electrochemistry ... 10
3.2 Nanoimpact experiments ... 10
3.3 Determination of the electrochemical active area of Au microelectrode and Pt ultramicroelectrode ... 10
4 Control experiments ... 11
5 Identification of the current spikes ... 12
6 MB-Monolith interaction and the expected charge of a single MB-Origami impact event ... 12
6.1 Case I: MB intercalates between base-pairs ... 13
6.2 Case II: MB molecules interact electrostatically with negative charged residues of DNA ... 13
6.3 Total number of MB molecules interacting with the monolith ... 14
7 Estimation of the MB/Origami ratio and the expected charge of a single MB-Origami impact event ... 14
7.1 Surface density of Monolith ... 14
7.2 Surface density of MB molecules ... 15
7.3 Calculation of MB molecules per origami structure and expected spike’s charge ... 16
8 References ... 16

FIGURES

Figure S1. DNA Origami design ... 7
Figure S2. TEM characterization of the Monolith. ... 8
Figure S3. AFM characterization of the Monolith. ... 8
Figure S4. UV-Vis spectra of DNA samples ... 9
Figure S5. Agarose gel of DNA samples ... 9
Figure S6. Determination of the electrode’s area. ... 11
Figure S7. Control CA traces. ... 11
Figure S8. Spike identification ... 12
Figure S9. DNA-Methylene blue interaction ... 12
Figure S10. Surface density of Monolith. ... 15
Figure S11. Surface density of MB molecules. ... 16

1 Synthesis and purification of Monolith

1.1 Staple list. The staple sequences for DNA Origami monolith assembly are listed below.

AAT TGC TCC TTT TAA GAT TAG TTG CTA TTT TG
CTC GTA TTT AGA GCC GTG GAA GGG AAC AAT AAT ATT CAT TTC AAT TAC
TTT AAA TAG TTT AGA CGA GGC TTT
CGA AAG AGG CAA AAG TTA CTT ACC GAT ATA TTC GGT C
TTT AAA CTA CAA CGT TGA AAA TCT CCA TTT
GGA CTC CAC GAA AGG AGC GGG CGC TAG GGC G
TGG GGT GCC TAA TGA GAC CGT CGG GGT AAA GG
TTT TCG CGT CCG TGA CTC ACA TTA ATT GTT T
TCT GTC CAT TCC TTA TCA CTC ATC GAG AAC A
GCT CCA TGA ATA CAC TAC TTT TTC ATG AGG A
TTT TCA GAG AGA TAA CAC TGG CAT TTT
AGC CAT TTG GGA ATT AGA ATG GAA AGC GCA
TTT AGA AAA CTA ATA GAT AAG TCC TTT
AAA GTA CCG ACA AAA GGA TAA AAA TTT TTA GA
GCG CAT AGC GCG ACC TTA AGA ACT
TTA CAG GTA GAA AGA TAC CCC CAG AAA TTG TG
TAG GAG CAC TCA AAT ACC AGC AGA
GGT TGT GTG AAG GGT AGC CAA GCT
AAC AAC CCG TCT TTT TTT
TTT AGA ACC CTT TGA GGA TTT AGA TTT
GTT ATA TAA TTT AAT GAC ATA AAT AAG AAG ATG ATG AAA CAA ACA TC
TTT TTG AGG GGG TAA TAA GCC CGA AAG ACT TT
CTC AGG AGG TTT AGT ACC GCC ACC CTC AGA ACC GCC ACC
TTT TGA CGA CTG CGC GCC TGT GCA GAT CCA GCG CAG TGT CCT CAA TCG
ATC TAA AGC ATC ACC TTC GGG AGA TTA GAA CC
TTA TCC TGA ATC TTA CAG GCA TTT CAA CAT GT
TGA GCT AAG CCT CCT CTT GCA GCA AAA TCC TGT TTG ATG GTG GTT CCG
TTT GGA TTC TCC GTA CCA GGC AAA GCG TTT
TAA GCA GAT ACC GAA GCC CTT TTT TAC GAG GC
TTT TTG CCC CAG CAG GCG AAG CGG TCC ACG CTG GTT TT
AGA GCC AGC AAA AAC CGA ACT GAC CAA CTT TG
ACC TTC ATC AAG AGT ACC TTT AAT GCC GCT TT
GGG TAC CGG TAA AGC CGT CGT GCC
CTG AGC AAC AAT ATA TTA CCA TAT CAT ATT CCT GAT TAT C
CGG AAT ACT ACG CAG TCA AAG TCA TTT ATC CCC TTT CCA GAG CCT AAT
GAG AGG CGG TTT GCG TAT TGG CGC TGC GCG TA
GAC GGT CAA GGC ACC AGT AAA ATA CGT AAT GCA ATC ACC G
CTG CCT ATT TCG GAA CCT ATT GGG ATT TTG CTA AAC AAC TTT CAA CA
GCA AAT TAA CCG TTG GAC AGG AAT GCG CCG C
AGT TAA AGT GTA TCG GAT TGC GAA TAA TAA TTT TTT CAC G
TAC AGG GCG CGT ACT ATG GTT GCT AGG AGG CCG ATT AAA G
ATC AAT TGA CCC TGG AGC TTA ATT GCT GA
GAC GAC GAA CCG TGC ATA ACG CCA GGT GAA GG
TTT ACG AGT GCA GAA CTA TTC TAA ACG AGC GTA ATC CAA ATA AGA AAC
CGT TTG CCA TCT TTT CAT AAT CAA CAC TAC GA
AAG TTA AAG GAC TTG TTC CCA CGC
TGC AAC TAA CAG TTG AGG CAA GGC AAA GAA TTA GCT TT
CTT GCC TGG GAA AGC CAG CCC CCG ATT TAG AGT TT
TTT AAA GCC GCA CAG GTC AGC AGC AAC TTT
ACC AGA AGG AGC GGA TAG TGA AAA ACA GT
GCC TCC CTG TTG AGG CGG CCG GAA CCG ACT TG
GGG GTC AGA CAT GGC TTT TGA TGA TTG ACG GA
TTT GAA GGT AAA TAT ACA GGA GTG TTT T
TGC GGG ATA CGA GGG TCC AAG CGC GAA ACA AAG TAT TT
AGC GCC AAA AAC GTA GAA TGA AAC TCG GCA TT
GTT TCA GCA AAC AGC TTG ATA CCG
TCC ATC ACC ATG GAA AGG ATT ATT TAC ATT G
TTT GAG AAT CGC CAT TGC CAG TTA CTT T

GTA ATC AGC TCC TTA TCC AAA AGA CCA CAA GAA TTG AGT T
CAT AAT TAC TAG AAA AAG CCT GTT TAG TAT C
ACG TCA CCA AAA TAC AGA AAC CGA
ATA TCC AGA ACA ATA TAT TAG TAA AAG AAT ACA GGC GGT C
CGG ATT CGC CTG ATT GCA GCA GCA TAA TAC AT
TTC GTA ATG CGG CGG GGC CAG CGG TGC CGG T
TTT AAG TTA CAA AAA GGG CTT AAT TTT T
TAA CAT CAA GCT AAA CTT GAC GAG CAC GTA TAA CGT GCT TTC CTT T
TTT AGT ATT AGA CTT TAT CCT GAT TTT
GGG AAG GGC GAT CGG TGC GGG CCT AAC GTG CCC GAT GCT GAT TGC CGT
ATT TAA ATC GGT AAT CAA TGG GAT CCA GCT TT
TTT GAT TAA GAT AGC GAC AGA ATC TTT
CTC TGT GGT GCT GCG GCC AGA ATC ATG GTC GGT ATG AGC CGG GTC
CCA CCA CCC TCA TTT TCG TTT TGT CGT CTT TCC AGA CGT T
GAA GCC TTT GGG GCG CAA CCT GTT TAG CTA TAA TTA AAC C
TCA GTT GGC AAA TCA ATC GCT GGC
ACC ACC ACA CCC GCC GCG CTT AAC GGT ACG CCA GAA TC
TTT CTT GAC GGA GTA GAA GAA CTC TTT
AAC ATT ATC TAC TAA TGA CCA TTA GAT ACA T
GCA TCG TAC AGT ATC GGC CTC AGG AAG ATC G
ATC ATA AGG GAT CAC CAG TAG CAC CAT TAC CA
AGC GTC ATT GCC TTG AGT AAC AGT GCC CGT AT
CAT TAA AAA TAC CGA ACG AAC CA
ATT AAA GGT GAA TTA TTT CCA GTA CCA GCA TT
AGT TTC CAA CGC ATA AGC CGG AAC
CGA CGA TAA AAA CCA ACG AGT AGA CTG TAG CT
ATA GCT GTC CAC ACA ACG CGC GGG
AGC AAC GGC TAC AGA GCC ATG TAC GAC AGC CC
GAA CGC GAA ACT GAA CTA ATA AGA GGA AAC GCA ATA ATA A
CTG ATA AAA TTC AAA AGG GTG AGA CGA GCT TC
TTC ACA AAC AAC AAT GAC AAC AAC
CTG AAT AAT CAA TAG AAA TGA AAA
TTA ACG TCA TAT CTG GAG GAA GGT TAT CTA AAA TAT CTT
ATA GCC CGT CAC CAG TGA GAC GGG CAA CAG C
TAA GAG GCT GAG ACT AAA CAG TTA ATG CCC C
TTT AAA AAA AGG CTC CAA AAG GAG ATC TTG ACT GTA TCA T
TTT GGA ACT GTA GCG GTC AGC GCC AGG GTG GT
AGC GGG GTC ATT GCA GGC GCT TAA AAC ATC GC
TAT TTA ACT TAT CAA CTT TTC AAA CAA GAC AA
AAG GCC GGA GAC AGT CAA ATC TTT
GAC AAT ATA CCA GTC AGC CAT TGC AAC AGG AAA AAC GCT
ATT CGC GGA TGG GCG AGC AAA CAA GAG AA
TTT TCA TTA CCC AAA TCA ACG TAA TTA AGA GGA GTA AAA T
ATT ACC GCT TAT CCG GGC GCC TGT AAC GCC AA
ATG TGC TGC AAG GGA TTT TCA GGT
ATA GTT GCG CCG AAT AAA TCC TCA TTA AAG CC
GCC AAC GCT CAA CAG TTC GCG CAG AAA TCG TCT TAT ACT TTC AAT ATA
TTT AAT GGT TTA TCA ATC CGG CTT AGG TTG G
AAT CAT AGG TCT GAG ATT TTT TAA CCA ATA GG
AGC ACG CGT GCC TGT TCT TTT TT
AGA TGA TGA TAG CTT AAT AAC CTT AGT TAA TT
TGC CGG AAG GGA ACA ATG GAG CCG CGG CGA AA
TTT TGT TTG GAG CTA TTA ATT AAT TTT
CAT CAA CAT TGA CCG TGT AAA ACT
CAT AAA GTA GCT CGA ATG ATT GCC AAA ATC CCT TAT AAA TCA AAA GA
ACA GTT GAT TTC TGC CCG TTA ACG GCA TCA GA
TAT CAT AAT TCG CAA AAT GGC TTA
CTC AGA ACA ATT TTC TGT ATA TTC TGA AAC ATG AAA GTA T
TCG AAA TCG CTG GCT GGT GAA TAA
TGC CCG AAC GTT ATT AGC AGA TTC TTT TGA AT
TGG TCA ATG AGC TGA AAA GGT GGC
ATT AGA GAG TAC CTT TAC CCT CAT TTG CGG GA

AAA GAG GAG AGG CGC ATT TAA TCA
CTG AGA AGA GAG TCT GCT GGC AAG AAG AGT CCA CTA TTA AAG AAC GT
TTT CAA CGG AGA TTA AGA ACC GGA TAT TTT
ACC CTG AAA TGT TAG CAG ACA AAA
CCT GTA GCA TTC CAC ACG TAA CAC TGA GTT TCG TCA CCA GTA CTT T
TAA GTA TAC ACC CTC AAC CAC CAG
ATA AAA AAA TCC CGT AAA ATT TTT
CCC TCG TAA TAT TCT ACC TTA TGC GAT TT
ACC AGA AGT ACA TAA AAT TAG ACG GTT TAA CG
CAC AAT CAA TAG AAA ATT CAT AT
AAC GCG GTG CCA GAG CCC AGC ATC
TTT AAG TTT GCC TTT AAC CCT CAG TTT
TCG TCT CGC AGT TGA AAG GAA TTG
TCC AAT AAT AAA GCT ACA ACA TGT AAA GCG AA
TCG ATG AAT GTA AAC GTC CTG TAG CCA GCT TTT ACC AGT CCG GCC AGT
TGG GAA GAC AAC ATT ATG CAA AAG AAG TTT TGC CAT TT
GTA ATA AGA GAA TAT CAC CCA GCT ACA ATT
GGT CAG GTA CCC TGA CTA TTA TAG TCA GAA
CAG ATG AAC GGT GTA CAG ACC AG
AGT TGG GCT GCC GGG TCC TGC GGC
ATC AAG GGG GCG AAC GTG GCG AGA AAG GAA GG
TTG TTA AAT CAG CTC AGA CTA CCT TTT TAA CC
GCA AGA AAC AAT GAA AAG CAA GCC AGC GAA CC
GAA CCA GAG CCA CCA CCG GAA CC
CTT CTT TGT ACC GCC ACA CGA CCA GTA ATA AA
TTT ACC ATC AAT ATG ATA TTC AAC
AAG AAA ACT TAC CTT TGA AAT AAA AAG AAA CC
GTG AGT GAG ATT AAG ATA AAT GCT GAT GCA AA
AAA ACA GGA GCT ATC TTA GCC GAA CAA AGT T
CCG GCA CCG CTT CTG GCG TTC TAG AGC ATG TC
TTT TTT CCC TTA GAA TAG AAC GCG TTT
AGT ATA AAC ATG TAA TTT AGG CAG CAA CGC TA
TTT AAA ATA AAC AGA TTG AGG GAG GTT T
CTG TAG CGC GTT TTC ACA TCG ATA AGC CGC CG
GGA GTG AGA ATA GAA AGG AAC AAA GCG TAA C
GCA GCA CCA ATT ATT CGG GCG ACA TTC AAC CG
TCA AAC CCT CAA TCA AGA TGA ATA TAC AGT AAC AGT AC
CAC TCC AGA GGT CAC GTA AAA CGA CCG GAA TTT GTG AGA GAT AGA CT
AAG GCT ATC AGT AAA CAC CGG AAT
AAT GCA ATG CCT GAG CTC CAA CA
ATC ATA CAT TCC CAA TTC TGC GAA
ATA GGA ACG CTT TGA GTT GCA GGG
TTA GTG ATA CAT CGA CCG TAC AGC GCC ATG TT
TCA AAA ATG AAA ATA GCA GCC TTT ACA GAA ACG AGA ATG ACC ATA AA
TCA CAA TTT TCC TGT GTT TTC TTT AGA TAG GGT TGA GTG TTG TTC CAG
GAA CGG GTT TTT CAT TTA TTT CAA CGC AAG GTA AAG TAA T
TTG GTG TAT CTG GCC TTT AAT ATT TTG TTA A
TTT TTC AAA TAT CGC GTT TTA ATT
ACG GCG GAT TAA ATG TGA TTG TAT AAG CAA AT
AAC AGT TCA GAA GAG AAT AAC ATA
AGA ACG TCA GCG TGG TCA ACT GTT TTC AGA GG
TTC TTA CCT CAT CTT CTC AGC TAA CAT GTA GA
CCA TAT TAG AGG GTA ATA GAA GGC GCC CAA TA
TGA AAT TGG AGG CCA CCG AGT AAA TGT TTT TAT AAT CAG TTT ATC CGC
TTT ATC AGA TAT TGA GCG CTA ATA TTT
TTT AAA CTA TCG GCC TAC AGA GAT TTT
ACA AAC AAT TCG ACA AAG GGA CAT GAA AGC GT
AAT AAT CGG CTG TCT GAC GAC GTC CCG ACT TGC GGG A
AGC TGC ATT AAT GAA TCG GCC AAC ATA CGA GCC GGA AG
CCT TGA AAA CAT AGC GGC AAT TCA
TAG CAA TGA AGC GCG GTG GCA ACA TAT AA
AAA GGG GGA GCC TCC GCC GTT TTT

GCT GGT CTG GCG GCC TTT TCT TTG CAG CAA AT
TTT TGA ACA AGA AAA AGC AAG CAA TTT
TCG TCA TAT TAC CAG AAG GAA TAC CAC ATT C
TGG GCT TGA GAT GCG GAA TAA GTT TAT TTT GT
TCC GGC ACC AGT CAC GAC GTT G
AAT AGC GAT GGA TAG CAT ACC AGT CTC ATT CA
CAG ATA CAT AAC GCC AAA AGG AAT AAG AAA AG
TAA TAC TTA TAT TTT AGA TCT ACA
TTT CGC AAG AAT GCC AAC GGC AGC
ATC ATT TTA AAG AGA CGC AGA AAC
AGT ATT AAC ACC GCC TAG AGC GGG
AAG AAA CGC AAA GAC ACC AGT TTA ATT TCA AC
ACA ATA AAT CGA GCC AAT ATG CGT TAT ACA AA
GTC TCT GAA TTT ACC GCA CCG TCA
GAT AGC TCT CAC GGA AGC GGA ACA GAA ATT GCG TAC GAT TAA GTT GGG
TTT TCG TTA GAA TCG CAA CAG TGC CTT T
TAA TAT CCC ATC CTA ATC CAA TCG TAT ATT TTG CTT CTG TAG GCG AAT
AGA ACG AGT AGT AAA TTC AAA AAT CCC CTC AA
TAG CCC CCT TAT TAG TTA GCA AAG GTC AGA CGA TTG GCC TTG ATA
ACG GTC ATA CCG GGG GGG ATC CCC TGG TAA TGT GGT GCC A
TTT ACG CTG AGA GCC TTT GAA TAC CTT T
AAC TAA TGT TGT GAA TAT TGA ATC CAG GTC TT
AGC GGA TCA AAC TTA AGC CCC CTG GCG GTT GC
ACA TCC TCA TAA CGG CTT CGC TAT TAC GCC AGC TGG CG
CTT TTA CAT GCT GAA CCT AAC AAC
CCA GAC CGG AAG CAA ATA ATG TGT GTA CCA AA
CTG CGG AAA TAT AAT GTT TAG TTT AGT AGT AGC ATT AAC A
TTT ACT GGT AAT AAG TTT TAA CTG CTC AGT
TTT CGT TGC GCT CAC TGC CCG CTT TCC AGT CGG GAA ACC T
TTT CCT CAC CGG AAA CAA TCC ACG GGA ACG GAT AAA TAA CAG GAT TAG CAG AGC GAG G
TTT AGT TTC ATT CCA TAT AAA GTA CGG TGT CTG GAA TAA CAG GAT TAG CAG AGC GAG G
TTT CTT ACA CTG GTG TGT TCT CGT CAT AAA CAT CCA TAA CAG GAT TAG CAG AGC GAG G
TTT AAA CGA ACT AAC GGA AAA AAT CTA CGT TAA TAA TAA CAG GAT TAG CAG AGC GAG G
TTT TCA GAA AAG CCC CAA AGT ACC CCG GTT GAT AAA TAA CAG GAT TAG CAG AGC GAG G
TTT GAA AGA CAG CAT CGG ACG TCA CCC TCA GCA GCA TAA CAG GAT TAG CAG AGC GAG G
GGT TTT GAA AGT ACC GCA TTC CAA TCC GAA CTT GGC ATG CAT
AGT AAA TGC GCC ACC CTC AGA GTC CGA ACT TGG CAT GCA T
GGC TAT TAG TCT TTA ATG CGC GAT CTG AAA TTA CCT ACA TCC GAA CTT GGC ATG CAT
GAC AGG AGC AGA GCC GTT CGG TCA TCC GAA CTT GGC ATG CAT
AAT CAT ATA ACA GGA AGA GCG AGT TCC GAA CTT GGC ATG CAT
TCT GAC CTT CTG GCC ATG CTG GTA TCC GAA CTT GGC ATG CAT
ATG CTT TAA TAG TAA GAG CAA CAC TCC GAA CTT GGC ATG CAT
GAT CTA AAA GGG ATA GCA AGC CCA TCC GAA CTT GGC ATG CAT
CCA CCC TCA GAA CCG CGC CCG GAA TAG GTG TAT CAC CGT ATC CGA ACT TGG CAT GCA T
ACT GTT GCT ACC TGC ACC GTT TTC TCC GAA CTT GGC ATG CAT
TTT GTG CCG TCG AGA GGG TTG ATA TCC GAA CTT GGC ATG CAT
CCA GAA CCG AGC CAC CGC GTC AGA TCC GAA CTT GGC ATG CAT
GGC GTT TTG TTT TTA TAA CCA ATC TCC GAA CTT GGC ATG CAT
ATT TCT GCT CAT TTG CCG CCA GCT CCG AAC TTG GCA TGC AT
CAT CGC CCT TAA ACG GAC CTA AAA TCC GAA CTT GGC ATG CAT
ATT TTA AAA GTT TGA GTA ACA TTT CCG AAC TTG GCA TGC AT
GCT GAG GCG ACT AAA GAA AAC ACT TCC GAA CTT GGC ATG CAT
TGA CCT AAA CTA TAT GCG CTG AGA TCC GAA CTT GGC ATG CAT
AAT TCG CAT TAA ATT TAA CGC CAT TCC GAA CTT GGC ATG CAT
TAC GAC AGG TCC TAC TAG TAC AGA TAA AAC AGA GGT GGT GGC ACA TAA TAG ATA AAT CCT T
TAC GAC AGG TCC TAC TAG TAC GGC TTG CCC TGA CGA GAA ACA CC
TAC GAC AGG TCC TAC TAG TAC GGT TTA CCG ATT TTT TGG AGA ATT
TAC GAC AGG TCC TAC TAG TAC AAC CAG CTT ACG GCT GGA GGT GT

1.2 DNA Origami design.

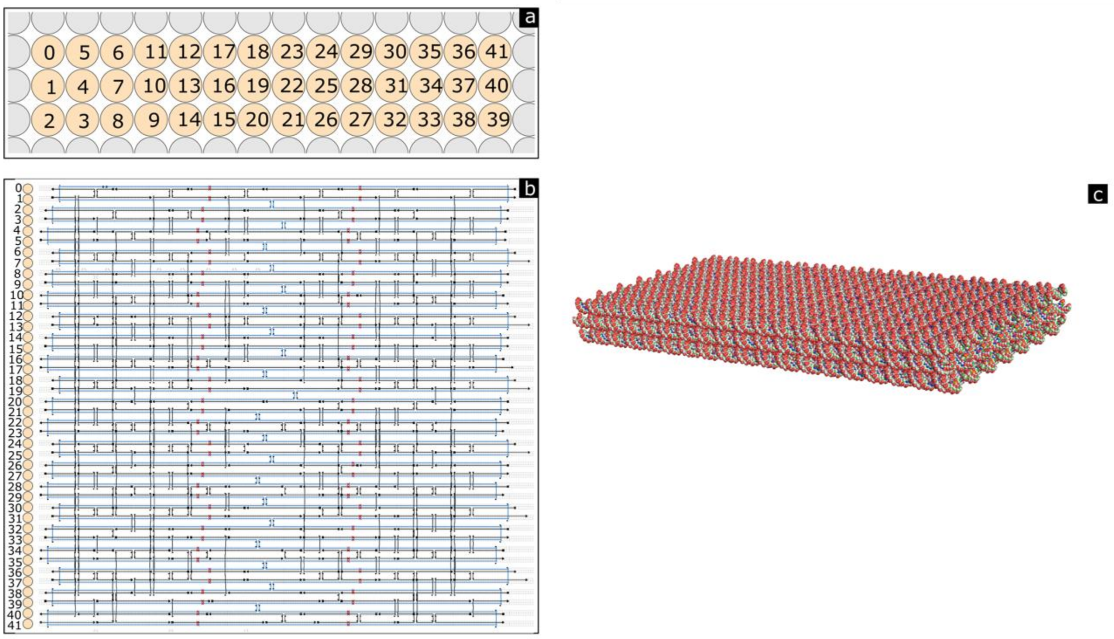

Figure S1. DNA Origami design. (a) 14×3 helix-bundle cross-section. (b) CaDNAno file showing structural design details of the DNA origami structure. The routing of the scaffold is marked in blue. The staple strands are shown in black. (c) PDB Atomic representation of the monolith DNA Origami structure visualised with Pymol [1].

1.3 Annealing and purification. 50 nM of p8064 DNA scaffold (provided by the Hendrik Dietz laboratory (TUM)), a staple mix containing 380 nM of each DNA staples (Integrated DNA Technologies) were mixed in a folding buffer (FoB) containing 5 mM Tris, 0.5 mM EDTA, 20 mM $MgCl_2$ and 5 mM NaCl , pH 8. Mixtures were annnealed from 80 ºC to 25 ºC over 16 hours (linear temperature ramps: 80 ºC to 65 ºC, 1 ºC min^{-1}, 65 ºC to 25 ºC, 2.5 ºC min^{-1}). The resulting annealed origami structures were purified using PEG purification method adapted from Stahl et al. [2]. Briefly, the sample was diluted to 500 µl with FoB buffer and mixed thoroughly in a 1:1 ratio with PEG precipitation buffer (1xTAE, 1 M NaCl, 11% w/v PEG8k), and centrifuged at 20 ºC with 20,000 rcf for 30 minutes. Afterwards the supernatant was carefully removed, and the pellet resuspended in TBE buffer. The concentration of Monolith was measured by UV-Vis spectroscopy (NanoPhotometer Pearl, Implen GmbH) considering an extinction coefficient of 0.13 $nM^{-1}cm^{-1}$.

2 Characterization of Monolith

2.1 TEM. TEM images were acquired with a FEI Tecnai T12 microscope operated at 120 kV with a Tietz TEMCAM-F416 camera. The Origami nanostructures were deposited on a glow discharged formvar coated carbon grid (FCF400-CU 400 Mesh, copper, Electron Microscopy sciences) using a negative staining protocol. 1 ul NaOH was added to a 100 ul 2% uranyl formate solution. The staining solution was centrifuged for 30 min at 20,000 rcf to avoid stain crystals. 5 ul of origami sample were deposited for 30 s. Subsequently, the grid was washed with 5 ul of staining solution, incubated for 40 s with 15 ul staining solution and dried with filter paper.

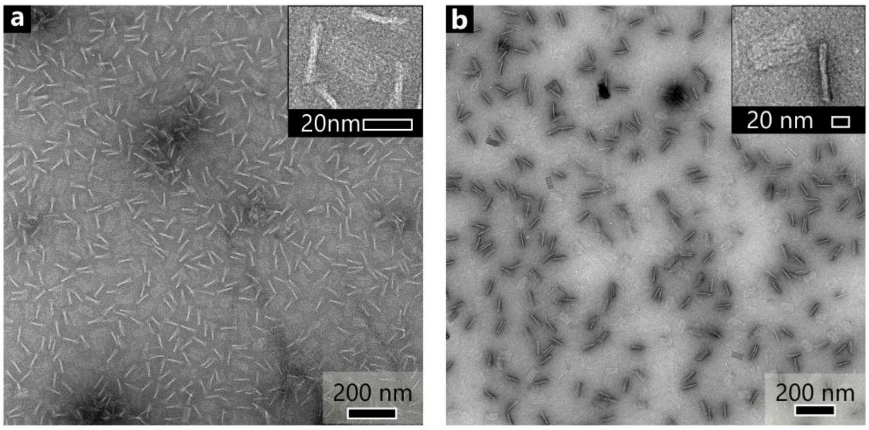

Figure S2. TEM characterization of the Monolith. (a) TEM image of the as-synthetized monolith. (b) TEM image of the monolith-MB samples used in nanoimpact experiments. Average dimensions: 57 nm × 28 nm.

2.2 AFM. AFM images were acquired with an Asylum Research Cypher ES (Oxford Instruments, UK) using Olympus BL-AC40TS-C2 (Olympus, Japan) cantilevers in AC mode. Monolith sample was deposited on freshly cleaved mica and imaged in folding buffer. AFM raw data was analyzed using WSxM software (Nanotec Electronica S.L., Spain[3]).

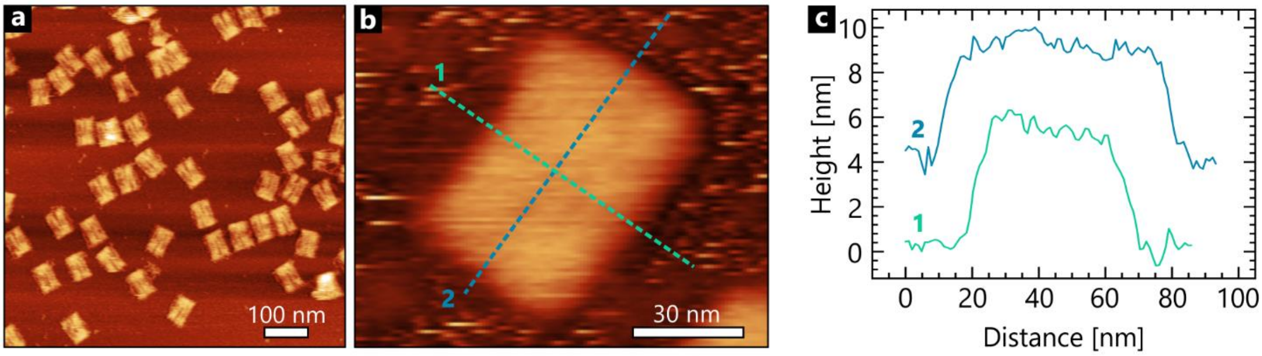

Figure S3. AFM characterization of the Monolith. (a) AFM image of the monolith on mica. (b) High resolution images of a single Monolith. (c) Height measurements (≈ 6 nm) along the indicated lines are shown. Height profiles are shifted for clarity.

2.3 UV-Vis. Measurements were taken using a JASCO v750 UV-Vis spectrophotometer and a low volume cuvette. UV-Vis spectra of the samples used for nanoimpact experiments are shown in Figure S4a. MB solutions are characterized by four peaks at 246, 292, 613 and 664 nm. The intensity ratio below to 1 between peaks at 613 and 664 nm rule out the presence of higher-order MB aggregates, typically observed for concentrated aqueous solutions [4]. b) A red-shift of ca. 3 nm in the peak at 664 nm is only observed for samples containing MB and monolith.

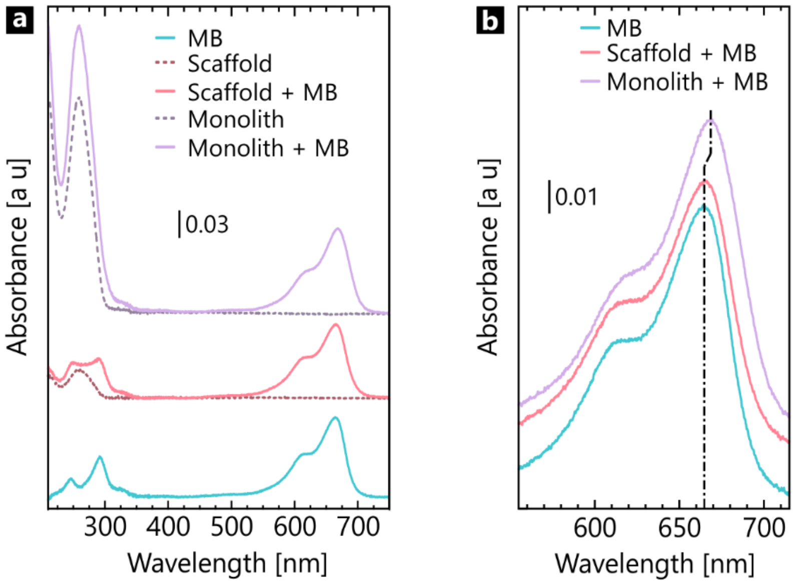

Figure S4. UV-Vis spectra of DNA samples. (a) Spectra of scaffold and monolith with and without MB. MB spectrum is also included. (b) MB signal shows a red-shift in peak wavelength only upon interaction with Monolith. [Scaffold] = 10 nM; [Monolith] = 20 nM and [MB] = 10 µM. Spectra were recorded in TBE buffer at 25 °C.

2.4 Agarose Gel Electrophoresis.

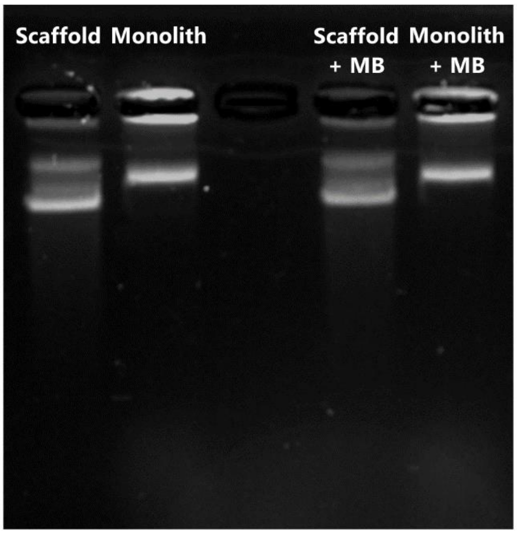

Figure S5. Agarose gel of DNA samples. Agarose gel (1.2 % w/v agarose) electrophoresis of scaffold and assembled monolith structures without (left) and with methylene blue (right). The gel was cast and run for 2 h at 3.5 V cm^{-1} in 45 mM Tris-borate, 11 mM $MgCl_2$, 1 mM EDTA, pH 8.3.

3 Materials and Methods

All chemicals were used as received. Source and purity are indicated in parenthesis along the text. Aqueous solutions were prepared using ultra-pure water (H_2O, 18.2 MΩ cm, Arium®pro, Sartorius, Germany).

3.1 Electrochemistry. Electrochemical experiments were performed in a conventional three-electrode glass cell using a HEKA PG340 USB potentiostat (HEKA, Germany) fitted with an external preamplifier (EPA) for low noise current measurements < 100 nA. The electrolyte was deoxygenated thoroughly using nitrogen prior to use and a blanket of nitrogen was maintained over the solution during all experiments. All measurements were carried out at room (~25 °C). For all the reported values, the stated errors are (Bessel-corrected) standard deviations based on at least 3 independent measurements. Pt coil and a Ag/AgCl(sat) served as the counter electrode and reference electrode, respectively. Au macroelectrodes (IJ Cambria, UK) and Pt ultramicroelectrodes (PtUME, IJ Cambria, UK) were employed as working electrodes (WEs) in bulk electrochemistry and nonimpact electrochemistry, respectively. Prior to use, WEs were polished with diamond 1 µm and 0.05 µm alumina slurries (IJCambria, UK). Afterward, the electrodes were subjected to an electrochemical cleaning, i.e., 20 times cycled at 0.5 Vs-1 in 0.5 M H_2SO_4 (98%, Roth) between the metal oxidation and Hydrogen evolution reaction (HER) regions to remove remaining contaminants (between -0.5 and 1.75 V for Au macroelectrodes and, from -0.25 to 1.35 V for Pt microelectrode). See section 9.3 for more details.

3.2 Nanoimpact measurements. Measurements were carried out in TBE buffer containing 10 µM of methylene blue (MB, ⩾97.0%, Sigma-Aldrich). TBE buffer refers to 0.5xTBE buffer (Roth, composition: 50 mM Tris-Borate, 1 mM EDTA) + 12 mM MgCl2 (⩾99%, Roth) at pH=8. Chronoamperometry (CA) traces were recorded at -0.3 V using a sampling interval of 100 µs, a 100 Hz Bessel filter and post digital filter of 3 Hz. Prior addition of the origami, several CA traces in 10 µM MB-TBE were recorded (three at least). These traces do not show any noticeable feature rather than a constant current baseline - cf. Figure 4a in the main text. Afterward, an aliquot of the monolith stock solution was added into the cell. The solution was mixed, N_2 was bubbling in for 5 min and CA traces were recorded.

3.3 Determination of the electrochemical active area of Au microelectrode and Pt ultramicroelectrode. For Au macroelectrodes, the electrochemical active area was determined using the reduction charge associated to the formation of a Au oxide monolayer. Cyclic voltammograms of Au electrodes were recorded in 0.5 M H_2SO_4 at 0.1 V s^{-1} between -0.25 and 1.65 V (Figure 6a). The charge ascribed to the reduction of a Au oxide monolayer (Q_{Au}, grey highlighted area in Figure 6a) was determined by integration of the cathodic peak centered at 0.90V and used to determine the area of the electrode (A) according to equation (1)

$$A = \frac{Q_{Au}}{390\ \mu C\ cm^{-2}} \qquad (1)$$

For PtUME, the electrochemical active area was determined using ferricyanide ions ($Fe(CN)_6K_3$, 1mM, ≥99%, Sigma Aldrich) in 0.1M KCl (≥99.5%, Roth). The reduction steady state currents (i_{ss}, see Figure 6b) were determined from voltammograms recorded at 0.01 Vs^{-1} between 0.5 and -0.1 V. The PtUME radius was obtained from equation (2)[5] and with it the area of the electrode using equation (3):

$$r = \frac{i_{ss}}{4nFDC} \qquad (2)$$

$$A = \pi\, r^2 \qquad (3)$$

where r is the PtUME's radius, n is the number of exchanged electrons (n =1), F is the Faraday constant, D and C are the diffusion coefficient ($D_{Fe(CN)_6^{+4}}$ = 7.6 ×10^{-6} cm^2 s^{-1}) [6] and the concentration (1mM) of the ferricyanide ions, respectively.

Electrode's areas were determined to be 0.077 ± 0.008 cm^2 for Au macroelectrodes and, 81.0 ± 0.5 μm^2 for PtUME.

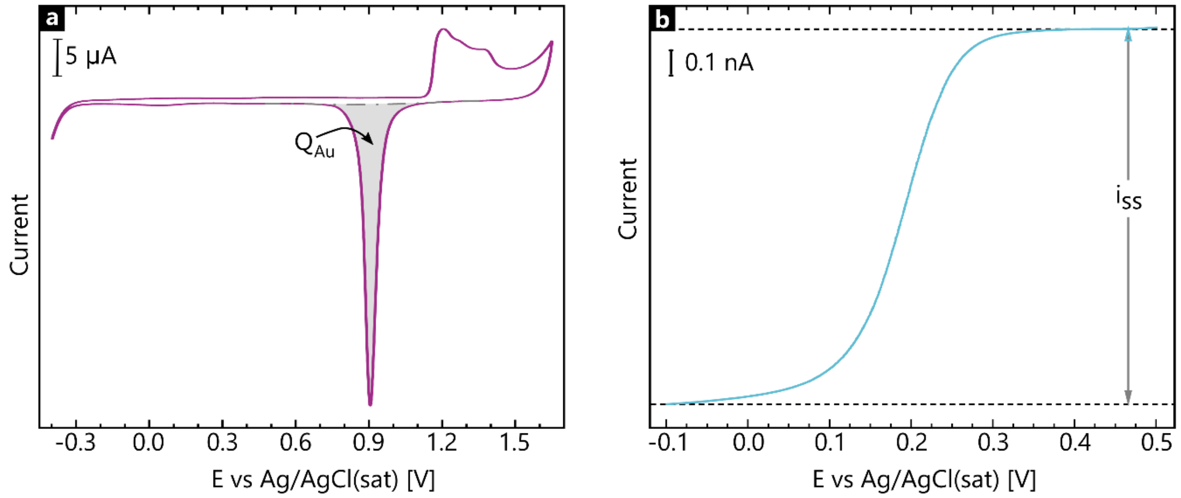

Figure S6. Determination of the electrode's area. (a) Typical cyclic voltammogram obtained for a Au macroelectrodes. The grey highlighted area is used to calculate the Q_{Au}. Scan rate: 0.1 Vs^{-1}, electrolyte: 0.5 M H_2SO_4. (b) Typical reduction current-voltage curve obtained for a Pt ultramicroelectrode. The reduction steady state current, i_{ss} is shown. Scan rate: 0.01 Vs^{-1}, electrolyte: 1mM $Fe(CN)_6K_4$ in 0.1 M KCl.

4 Control experiments

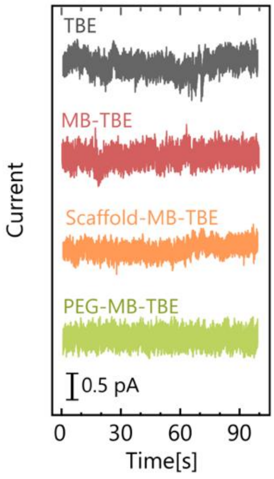

Figure S7. Control CA traces. TBE buffer, methylene-blue, scaffold, and PEG-MB at equivalent concentrations of the DNA Origami monolith did not show any significant peaks under same experimental conditions: at -300 mV vs Ag/AgCl in 10 uM MB 0,5X TBE +12 mM $MgCl_2$.

5 Identification of the current spikes

Spikes were identified with the peakfinder function in MATLAB[7], which uses the sign changing property of the first derivative of the signal to identify local maxima. Figure S8 shows a representative example of the resulting peak detection protocol (detected peaks marked with asterisks).

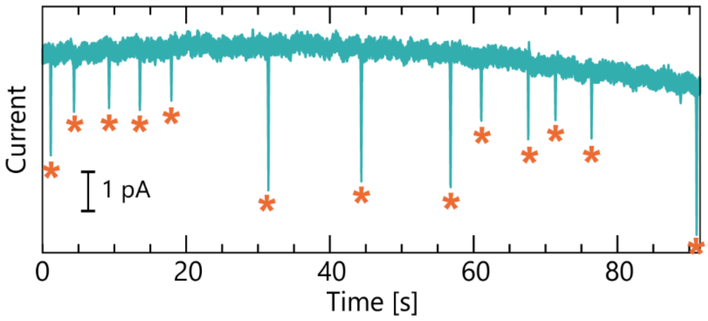

Figure S8. Spike identification Spike identification in representative nanoimpact signal. Asterisks indicate the detected peaks.

We then randomly sampled the identified peaks in 10000 replicates in samples sizes of 100 s duration to produce the histogram in Figure 3b.

6 MB-Monolith interaction and the expected charge of a single MB-Origami impact event

If methylene blue (MB) molecules are the only species involved in the charge transfer observed at -300 mV, the charge of a spike would be proportional to the number of MB that reacted, i.e., the number of MB molecules per monolith. Methylene blue (MB) can intercalate between bases of dsDNA and also interact electrostatically with the negatively charged residues of ssDNA. We consider the two scenarios here:

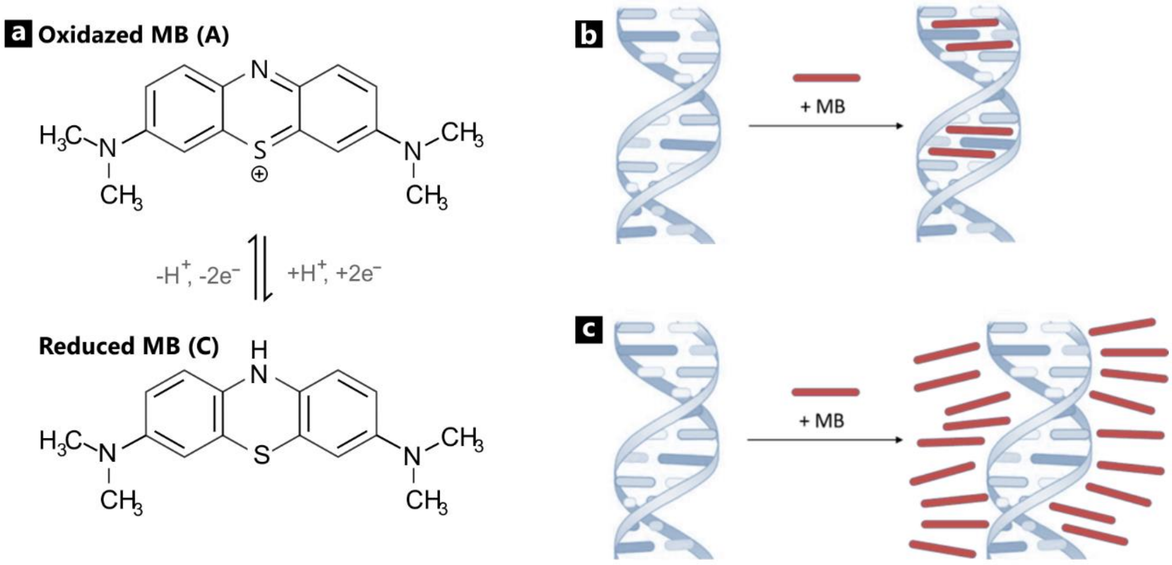

Figure S9. DNA-Methylene blue interaction. (a) Reduction and oxidation reactions of methylene blue (Image extracted from[8]). (b-c) Schemes of MB intercalated between DNA base-pairs (a) and, electrostatically MB interaction with negatively charged residues of DNA.

6.1 Case I: MB intercalates between base-pairs. MB intercalates between DNA base pairs, as has been widely reported in the literature [9, 10] (Figure S9b). We consider that the maximum MB molecules that can intercalate would be equal to the number of bases in the dsDNA. In a DNA origami this value is fixed by the scaffold strand. For the DNA Monolith structure considered in this the number of bases in the scaffold is 8064; hence the number of MB per monolith would be 8064.

According to Figure S9a, the electrochemical reduction of a single MB implies $2e^-$. Thus, the charge expected for a Monolith with MB molecules interacting only by intercalation would be:

$$Q_{MB\text{-}Monolith} = \frac{n_{total}\ F}{N_A} \qquad (4)$$

where n_{total} is the number of electrons exchanged per monolith, F is the Faraday constant and, N_A is the Avogadro's number.

$$Q_{MB\text{-}Monolith} = \frac{(2*8064)(96485.3329\ Cmol^{-1})}{6.023\times10^{23}\ particles\ mol^{-1}} = 2.58\times10^{-15}C \qquad (5)$$

$$Q_{MB\text{-}Monolith} = 2.58\ fC$$

The impact of a single monolith would lead to a spike with an expected charge of 2.58 fC. This value is 3 orders of magnitude lower than the experimental one.

6.2 Case II: MB molecules interact electrostatically with negative charged residues of DNA. It has been found that MB can interact electrostatically with negative charged residues of DNA[9-11]. If the negatively charged residues are only the phosphate groups, the maximum MB that can interact with the monolith would be equal to the number of phosphate group in it, as each phosphate has a charge equal to -1 and MB has a charge of +1 (see scheme in Figure S9b). As there is one phosphate group per base, the maximum number of MB that can electrostatically interact with the monolith would be equal to 2 times the number of bases in the scaffold, i.e.16128.

According to Figure S9a, the electrochemical reduction of a single MB implies $2e^-$. Thus, the charge expected for a Monolith with MB molecules interacting only by intercalation would be:

$$Q_{MB\text{-}Monolith} = \frac{n_{total}\ F}{N_A} \qquad (6)$$

where n_{total} is the number of electrons exchanged per monolith, F is the Faraday constant and N_A is the Avogadro's number.

$$Q_{MB\text{-}Monolith} = \frac{(2*16128)(96485.3329\ Cmol^{-1})}{6.023\times10^{23}\ particles\ mol^{-1}} = 5.16\times10^{-15}C \qquad (7)$$

$$Q_{MB\text{-}Monolith} = 5.16\ fC$$

The impact of a single monolith would lead to a spike with an expected charge of 5.16 fC. This value is 3 orders of magnitude lower than the experimental one.

6.3 Total number of MB molecules interacting with the monolith. From section 6.1 and 6.2, if MB-Monolith interaction is given by both intercalation and electrostatic interaction, the spikes ascribed to the impact of a single monolith would show a charge of

$$Q_{MB\text{-}Monolith} = 2.58\ fC + 5.16\ fC = 7.74\ fC \quad (8)$$

The value is 3 orders of magnitude lower than the experimental one, indicating that the two simple scenarios considered here are not representative of the real system.

7 Estimation of the MB/Origami ratio and the expected charge of a single MB-Origami impact event.

Au macroelectrodes were immersed in 50 nM origami solution in the dark for 30 min. Afterward, half of the electrodes were immersed for 5 min in TBE buffer (Au-Origami samples) and the others in 1mM MB-TBE solution (Au-Origami-MB samples) for the same period. Both kind of samples were rinsed with TBE buffer and dried with N_2.

7.1 Surface density of Monolith. The amount of origami on the surface was determined using the Au-Origami samples and the approach proposed by Steel and co-workers [12] (see Figure S10a). The redox marker can interact with the origami and thus being adsorbed on the surface or can be freely diffused to (from) the surface. This behavior is clearly seen in the cyclic voltammogram (Figure S10b), where the two scenarios lead to two different pairs of peaks (peaks 1 and 2, respectively. Importantly, the fact that ΔE_{peak} between peaks 2 is 60 ± 1 mV rules out that origami is blocking the charge transfer (ΔE_{peak} = 59 mV for an ideal reversible one electron transfer process, at 25 ºC).

The origami surface coverage was determined as follows: by using chronocoulometry, the electrode capacitances in presence and absence of the redox marker ruthenium (III) hexamine ion ($[Ru(NH_3)_6]^{3+}$, 50µM, 98%, Sigma Aldrich) in 10 mM Tris (⩾99.9%, Roth) are measured. The chronocoulombimetric traces are obtained using a pulse period of 1s and a pulse width of 0.65 V (applied potential step between 0.15 and -0.5 V, see Figure S10c for more details). The saturated surface excess of redox marker is associated with the origami surface density accordingly to equation (9) [12]

$$\Gamma_{origami} = \frac{zN_A\Gamma_0}{m} \quad (9)$$

where $\Gamma_{origami}$ is the origami surface density in molecules/cm^2, m is the number of bases in the probe origami (m=8064), A is the electrode's area, z is the charge of the redox molecule, N_A is Avogadro's number and Γ_0 the surface excess - i.e., the amount of redox marker confined near the electrode surface. The latter value corresponds to the difference in chronocoulometric intercepts for the identical potential step experiment in the presence and absence of redox marker (cf. Figure S10d).

Following the above approach, we found that $\Gamma_{origami}$ = (3.3 ± 0.2) ×10^{10} origami structures per cm^2, in good agreement with a surface fully saturated with origami structures in lying down configuration (the projected origami area is 1.6×10^{-11} cm^2, hence the expected $\Gamma_{origami}$ is 6.3 ×10^{10} origami structures per cm^2). This value does not change with the redox probe concentration indicating that 50 µM is enough to guarantee the saturation condition (Figure S10e).

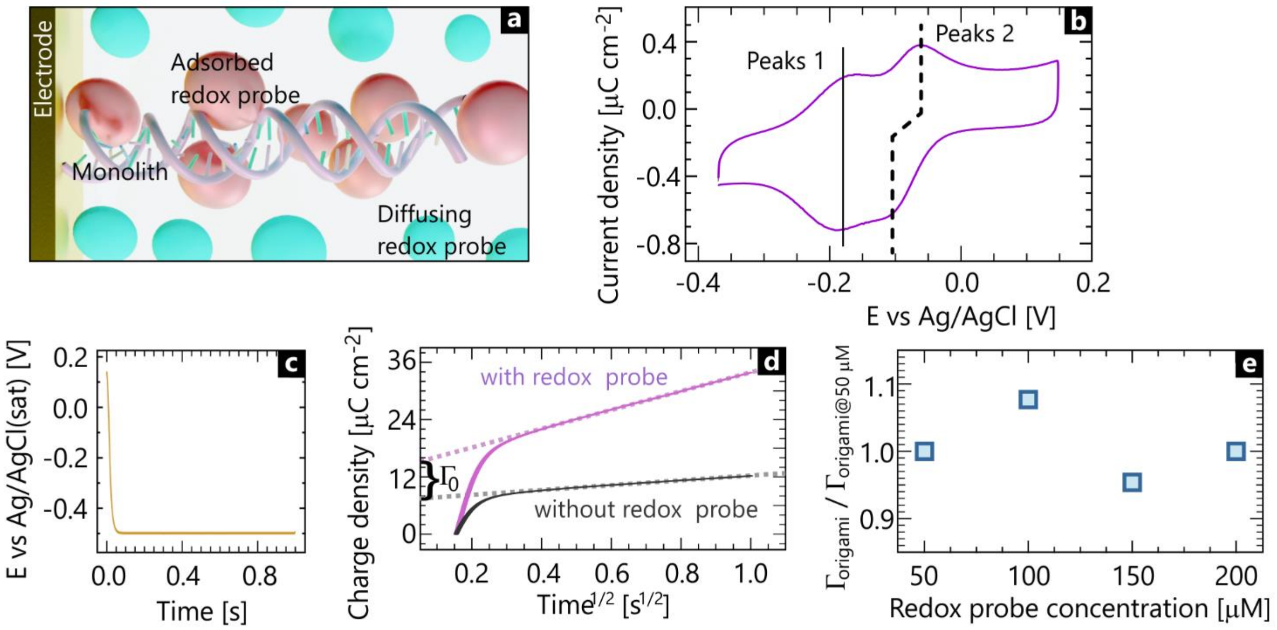

Figure S10. Surface density of Monolith. (a) Scheme of the redox probe adsorption when a Au electrode is covered by monolith structures. Redox probe can be adsorbed at the surface electrode by interaction with the negative charged monolith or diffuse to (from) the electrode surface. (b) Typical cyclic voltammogram of Au-monolith sample in 50 µM $[Ru(NH_3)_6]^{3+}$ in 10 mM Tris showing the two pairs of redox peaks ascribed to: adsorbed redox probe (peaks 1) and diffusing species (peaks 2). The lines highlight the ΔE_{peak}, which is almost zero for adsorbed species and 59 mV for free diffusion of the redox species. (c) Electrochemical potential profile used in chronocoulombimetric experiments. (d) Typical charge density response against the square root curve obtained by application of the above potential profile. Curves were obtained for Au-Origami samples without (black) and with (purple) the redox probe (50 µM $[Ru(NH_3)_6]^{+3}$ in 10 mM Tris). (e) Monolith surface coverage (relative to the value obtained at 50 µM) as a function of the redox probe concentration. The values lay between 10% indicating that 50 µM is enough to guarantee the saturation condition.

7.2 Surface density of MB molecules. Typical cyclic voltammogram obtained for Au-Origami-MB samples. A and C highlight the anodic and cathodic peaks, respectively. The grey highlighted area is used to calculate the Γ_{MB}, by using the peak charge MB (Figure S8) in the following equation:

$$\Gamma_{MB} = \frac{Q_{MB}}{\upsilon}\frac{N_A}{nF} = \frac{Q_{MB}}{\upsilon}\frac{q_e}{n} \qquad (10)$$

where υ is the scan rate, N_A is the Avogrado's number, F is the Faraday constant, q_e is the electron charge and n the number of electrons exchanged in the redox reaction (n = 2 for MB reduction, Figure S9a).

Following the above protocol, we found that Γ_{MB} = (10 ± 3) ×10^{12} MB molecules per cm^2.

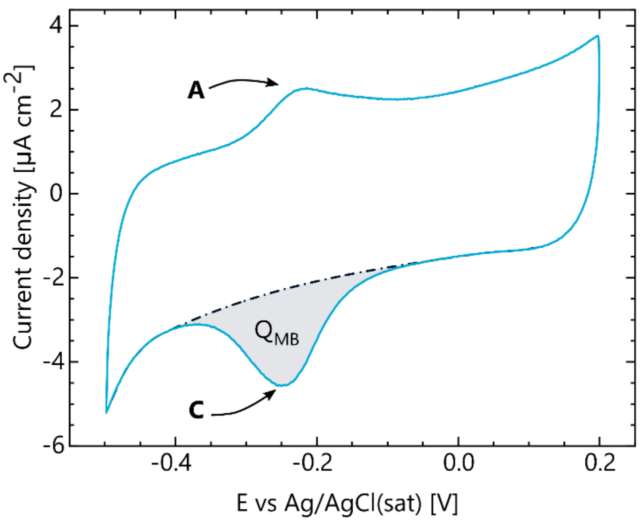

Figure S11. Surface density of MB molecules. Typical cyclic voltammogram obtained for Au-Origami-MB samples. A and C highlight the anodic and cathodic peaks, respectively. The grey highlighted area is used to calculate the Q_{MB}. Scan rate: 0.1 Vs^{-1}, electrolyte: TBE.

7.3 Calculation of MB molecules per origami structure and expected spike's charge. The number of MB molecules per origami structure ($x_{MB\text{-}Origami}$) is determined by equations 11,

$$x_{MB\text{-}Origami} = \frac{\Gamma_{MB}}{\Gamma_{origami}} \qquad (11)$$

And with it, the expected charge when all the MB molecules interacting with a single origami structure are reduced at surface's electrode (Q_{spike}, spike's charge)

$$Q_{spike} = \frac{nF\, x_{MB\text{-}Origami}}{N_A} \qquad (12)$$

We found that there are 307 ± 101 MB molecules per origami structure, hence Q_{spike}= (0.10 ± 0.03) fC.

8 References

1. The PyMOL Molecular Graphics System, Version 2.0 Schrödinger, LLC.
2. Stahl, E., et al., *Facile and scalable preparation of pure and dense DNA origami solutions.* Angew Chem Int Ed Engl, 2014. **53**(47): p. 12735-40.
3. Horcas, I., et al., *WSXM: a software for scanning probe microscopy and a tool for nanotechnology.* Review of scientific instruments, 2007. **78**(1): p. 013705.
4. Fernández-Pérez, A. and G. Marbán, *Visible Light Spectroscopic Analysis of Methylene Blue in Water; What Comes after Dimer?* ACS Omega, 2020. **5**(46): p. 29801-29815.
5. Zoski, C.G., *Ultramicroelectrodes: design, fabrication, and characterization.* Electroanalysis, 2002. **14**(15-16): p. 1041-1051.
6. Stackelberg, M., M.v. Pilgram, and V. Toome, *Bestimmung von Diffusionskoeffizienten einiger Ionen in wäßriger Lösung in Gegenwart von Fremdelektrolyten. I.* Zeitschrift für Elektrochemie, Berichte der Bunsengesellschaft für physikalische Chemie, 1953. **57**(5): p. 342-350.
7. Yoder, N. *peakfinder(x0, sel, thresh, extrema, includeEndpoints, interpolate)*. 2021; Available from: https://www.mathworks.com/matlabcentral/fileexchange/25500-peakfinder-x0-sel-thresh-extrema-includeendpoints-interpolate.

8. Silva, F.B., et al., *Electrochemical Investigation of Oligonucleotide-DNA Hybridization on Poly(4-Methoxyphenethylamine).* International Journal of Molecular Sciences, 2008. **9**(7): p. 1173-1187.
9. Farjami, E., et al., *DNA interactions with a Methylene Blue redox indicator depend on the DNA length and are sequence specific.* Analyst, 2010. **135**(6): p. 1443-1448.
10. Nafisi, S., et al., *Stability and structural features of DNA intercalation with ethidium bromide, acridine orange and methylene blue.* Journal of Molecular Structure, 2007. **827**(1): p. 35-43.
11. Kara, P., et al., *Electrochemical genosensor for the detection of interaction between methylene blue and DNA.* Electrochemistry Communications, 2002. **4**(9): p. 705-709.
12. Steel, A.B., T.M. Herne, and M.J. Tarlov, *Electrochemical quantitation of DNA immobilized on gold.* Analytical chemistry, 1998. **70**(22): p. 4670-4677.
